# Transcriptomic response of *Gordonia* sp. strain NB4-1Y when provided with 6:2 fluorotelomer sulfonamidoalkyl betaine or 6:2 fluorotelomer sulfonate as sole sulfur source

**DOI:** 10.1007/s10532-020-09917-8

**Published:** 2020-11-05

**Authors:** Eric M. Bottos, Ebtihal Y. AL-shabib, Dayton M. J. Shaw, Breanne M. McAmmond, Aditi Sharma, Danae M. Suchan, Andrew D. S. Cameron, Jonathan D. Van Hamme

**Affiliations:** 1 Department of Biological Sciences, Thompson Rivers University, Kamloops, BC V2C 0C8 Canada; 2 Department of Biology, University of Regina, Regina, SK S4S 0A2 Canada; 3 Faculty of Science, Institute for Microbial Systems and Society, University of Regina, Regina, SK S4S 0A2 Canada

**Keywords:** *Gordonia* sp. strain NB4-1Y, 6:2 Fluorotelomer sulfonamidoalkyl betaine (6:2 FTAB), 6:2 Fluorotelomer sulfonate (6:2 FTSA), Transcriptome, Per- and polyfluoroalkyl substances (PFAS)

## Abstract

**Abstract:**

Perfluoroalkyl and polyfluoroalkyl substances (PFAS) are environmental contaminants of concern. We previously described biodegradation of two PFAS that represent components and transformation products of aqueous film-forming foams (AFFF), 6:2 fluorotelomer sulfonamidoalkyl betaine (6:2 FTAB) and 6:2 fluorotelomer sulfonate (6:2 FTSA), by *Gordonia* sp. strain NB4-1Y. To identify genes involved in the breakdown of these compounds, the transcriptomic response of NB4-1Y was examined when grown on 6:2 FTAB, 6:2 FTSA, a non-fluorinated analog of 6:2 FTSA (1-octanesulfonate), or MgSO_4_, as sole sulfur source. Differentially expressed genes were identified as those with ± 1.5 log_2_-fold-differences (± 1.5 log_2_FD) in transcript abundances in pairwise comparisons. Transcriptomes of cells grown on 6:2 FTAB and 6:2 FTSA were most similar (7.9% of genes expressed ± 1.5 log_2_FD); however, several genes that were expressed in greater abundance in 6:2 FTAB treated cells compared to 6:2 FTSA treated cells were noted for their potential role in carbon–nitrogen bond cleavage in 6:2 FTAB. Responses to sulfur limitation were observed in 6:2 FTAB, 6:2 FTSA, and 1-octanesulfonate treatments, as 20 genes relating to global sulfate stress response were more highly expressed under these conditions compared to the MgSO_4_ treatment. More highly expressed oxygenase genes in 6:2 FTAB, 6:2 FTSA, and 1-octanesulfonate treatments were found to code for proteins with lower percent sulfur-containing amino acids compared to both the total proteome and to oxygenases showing decreased expression. This work identifies genetic targets for further characterization and will inform studies aimed at evaluating the biodegradation potential of environmental samples through applied genomics.

**Graphic Abstract:**

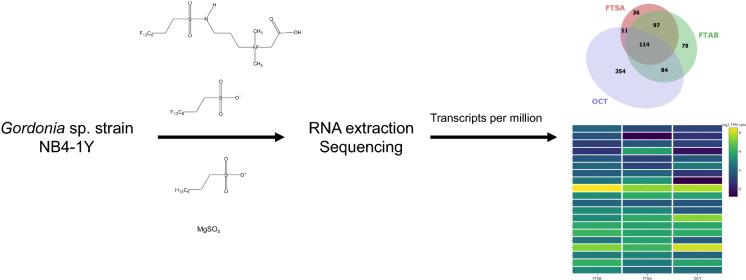

**Electronic supplementary material:**

The online version of this article (10.1007/s10532-020-09917-8) contains supplementary material, which is available to authorized users.

## Introduction

Perfluoroalkyl and polyfluoroalkyl substances (PFAS) represent a diverse group of anthropogenic compounds of concern due to their widespread use, potential toxicity (Borg et al. 2013; Piekarski et al. 2020; Rand and Mabury 2017; Stanifer et al. 2018), and resistance to complete removal from sewage (Choi et al. 2019; Dimzon et al. 2017; Lazcano et al. 2019; Stroski et al. 2020), drinking water (Hu et al. 2016; Li et al. 2020; Rahman et al. 2014), landfills (Hamid et al. 2018; Hepburn et al. 2019; Knutsen et al. 2019) and environmental reservoirs (Ahrens et al. 2015; Ahrens and Bundschuh 2014; Barzen-Hanson et al. 2017; Codling et al. 2020; Janousek et al. 2019; Ross et al. 2018). Much of what is known about microbial PFAS metabolism has been derived from chemical analyses of soil, water, groundwater and sediment, mass balance studies of sewage treatment systems (reviewed by Ahrens and Bundschuh 2014; Liu and Mejia Avendaño 2013), and in vitro microcosm studies using aerobic (D’Agostino and Mabury 2017; Liu and Liu 2016; Liu et al. 2010; Wang et al. 2005; Zhang et al. 2016) or anaerobic (Zhang et al. 2016) mixed cultures taken from these environments. Pure culture studies have detected similar suites of metabolic products from PFAS such as 4:2, 6:2 and 8:2 fluorotelomer alcohols, 6:2 polyfluoroalkyl phosphates, 6:2 fluorotelomer sulfonamidoalkyl betaine (6:2 FTAB) and 6:2 fluorotelomer sulfonate (6:2 FTSA) (Key et al. 1998; Kim et al. 2012, 2014; Lewis et al. 2016; Liu and Mejia Avendaño 2013; Presentato et al. 2020; Shaw et al. 2019; Van Hamme et al. 2013), although no studies have identified proteins involved in the generation of these metabolites.

Microbial community metabarcoding tools and quantitative polymerase chain reaction have been applied in an effort to correlate changes in microbial community structures to PFAS contamination in order to form hypotheses about the main microbial players involved in PFAS transformations (Bao et al. 2018; Fitzgerald et al. 2019; Ke et al. 2020; O’Carroll et al. 2020; Zhang et al. 2019a, b, 2020). O’Carroll et al. (2020) observed an increase in the relative abundance of *Gordonia* spp. at a decommissioned firefighting training centre in western Canada where aqueous film-forming foams (AFFF) for hydrocarbon firefighting had been used over 40 years, suggesting that this genus may be actively metabolizing PFAS in soil and groundwater environments. While multi-omic studies examining DNA, RNA, protein and metabolite profiles of microbial communities in PFAS-impacted environments will be valuable for guiding bioremediation, in order to make sense of these complex datasets, fundamental knowledge of the biochemical mechanisms of specific PFAS metabolic steps is needed.

We have focused on understanding the molecular biology underlying PFAS metabolism in the aerobic soil bacterium, *Gordonia* sp. strain NB4-1Y, with interest in desulfonation reactions that liberate sulfite from 6:2 fluorotelomer sulfonamidoalkyl betaine (6:2 FTAB) and 6:2 fluorotelomer sulfonate (6:2 FTSA) (Shaw et al. 2019; Van Hamme et al. 2013). The *Gordonia* genus is related to *Rhodococcus* and, as a group, these organisms have relatively large, GC-rich genomes that include a diversity of oxygenases and degradative enzymes allowing them to access an array of natural and anthropogenic organic compounds for energy and nutrients (Arenskotter et al. 2004; Brooks and Van Hamme 2012; Drzyzga 2012; Hara et al. 2007; Larkin et al. 2005; Martinkova et al. 2009; McLeod et al. 2006; Van Hamme et al. 2004).

6:2 FTAB is a key ingredient in some AFFF formulations, used in commercial, military, naval and aviation applications; in turn, the suspected environmental source of 6:2 FTSA is as a byproduct of 6:2 FTAB degradation (D’Agostino and Mabury 2017; Moe et al. 2012). Following AFFF use, these and other PFAS contaminated aquatic and terrestrial environments (Baduel et al. 2015, 2017; Dauchy et al. 2017; Munoz et al. 2017). NB4-1Y transforms both 6:2 FTAB and 6:2 FTSA to 16 observable metabolites over 7 days of laboratory incubation when the compounds are provided as sole sulfur sources (Shaw et al. 2019).

Here we examine the transcriptomic responses of *Gordonia* sp. strain NB4-1Y to 6:2 FTAB or 6:2 FTSA as sole added sulfur source in pure laboratory cultures. RNA sequencing (RNA-seq) enables the quantitative analysis of transcription across all genetic elements in a genome. This genome-wide perspective is useful for finding genes that are differentially expressed in response to compounds such as 6:2 FTAB or 6:2 FTSA. To find coordinated metabolic programs in NB4-1Y, global gene expression in the presence of 6:2 FTAB or 6:2 FTSA was compared to NB4-1Y cultures growing on either MgSO_4_ as a sulfate rich control, or the sodium salt of 1-octanesulfonate as a non-fluorinated structural analogue of 6:2 FTSA. Through these analyses we identified, for example: 20 genes associated with the transport and metabolism of sulfur compounds more highly expressed with 6:2 FTAB, 6:2 FTSA and OCT compared to MgSO_4_; three genes associated with carbon–nitrogen bond cleavage more highly expressed with 6:2 FTAB; three alcohol dehydrogenases, three monooxygenases, and three genes associated with acetyl-CoA metabolism, as being more highly expressed with 6:2 FTAB and 6:2 FTSA. These data are being used to identify target genes that will be cloned and expressed so that the activity of purified enzymes against 6:2 FTAB, 6:2 FTSA, and breakdown products can be characterized.

## Materials and methods

### Chemicals and culture media

The ammonium salt of 6:2 FTSA (Synquest Laboratories; 98.0% pure by NaOH titration), a technical grade solution of 6:2 FTAB (0.27 g mL^−1^; Shanghai Kingpont Industrial Co. Ltd., Shanghai, China), sodium 1-octanesulfonate and magnesium sulfate (Millipore Sigma, St. Louis, MO, USA) were prepared at 4 mM in a 50:50 mixture of anhydrous ethanol (Commercial Alcohols, Brampton, ON, Canada) and 18 MΩ water. The 6:2 FTSA solution was heated to 55 °C and stirred overnight to dissolve. All other chemicals were from Millipore Sigma.

Van Hamme et al. (2004) was followed for the preparation of liquid sulfur-free acetate (SFA) medium with, per liter: 0.40 g potassium dibasic phosphate (KH_2_PO_4_), 1.60 g potassium monobasic phosphate (K_2_HPO_4_), 1.55 g ammonium chloride (NH_4_Cl), 5.00 g sodium acetate (NaCH_3_COO), 0.165 g magnesium chloride (MgCl_2_), 0.090 g calcium chloride dihydrate (CaCl_2_·2H_2_O), 5.00 mL Wolfe’s minerals, and 1.00 mL Pfenning’s vitamin solution. KH_2_PO_4_, K_2_HPO_4_, NH_4_Cl and NaCH_3_COO were autoclaved in 18 MΩ water at 121 °C for 20 min, cooled to room temperature, and a 0.22 µM pore sized filter sterilized solution of MgCl_2_, CaCl_2_·H_2_O, Wolfe’s minerals and Pfenning’s vitamin solution was added aseptically. Difco Nutrient Agar was used for the preparation of solid medium (BD Biosciences, San Jose, CA, USA).

### *Gordonia* sp. strain NB4-1Y growth conditions

*Gordonia* sp. strain NB4-1Y was isolated from vermicompost for its ability to cleave subterminal alkyl carbon–sulfur bonds, and has been shown to metabolize 6:2 FTAB and 6:2 FTSA (Shaw et al. 2019; Van Hamme et al. 2013). Pure culture stocks are maintained on Microbank beads (Pro-Lab Diagnostics, Inc., Richmond Hill, ON, Canada) stored at − 80 °C and, prior to each experiment, a single bead is streaked onto nutrient agar and incubated at 30 °C until isolated colonies are visible.

Next, single isolated colonies from nutrient agar plates were used to prepare inoculum cultures in sterile 20-mL culture tubes containing 5 mL SFA medium and 75 µL of sulfur source stock solution (i.e., 4 mM 6:2 FTSA, 4 mM 6:2 FTAB, 4 mM OCT, 4 mM MgSO_4_, or a no sulfur added control consisting of 50% ethanol). Sterile controls for each sulfur source were also prepared. All cultures were incubated on a tissue culture roller drum (New Brunswick Scientific, Enfield, CT, USA) set to 150 rotations per minute at 30 °C.

Experimental cultures were prepared in sterile 125-mL Erlenmeyer flasks containing 25 mL SFA media with 375 µL of sulfur source stock solution (final concentration 60 µM) and inoculated with 250 µL of the respective inoculum culture (i.e. 6:2 FTSA inoculum culture used to inoculate the 6:2 FTSA experimental culture, etc.). These were plugged with foam stoppers and covered with aluminum foil, and incubated in an orbital shaker at 30 °C with 150 rotations per minute.

### Biomass collection and RNA stabilization

Once cultures reached the mid-log phase, 10 mL of freshly prepared 5% (vol/vol) phenol in absolute ethanol was added to each 25 mL culture in order to neutralize cellular activity and stabilize RNA prior to extraction. These were incubated on ice for 1.5 h and, following this, 1.4 mL aliquots were centrifuged in microcentrifuge tubes for 5 min at 8200×*g* to pellet biomass. Supernatants were removed, pellets flash frozen in liquid nitrogen, and shipped on dry ice to the University of Regina for RNA extraction and sequencing.

### Bacterial lysis and total RNA isolation

A number of methods were tested, with limited success, for extracting RNA from *Gordonia* sp. strain NB4-1Y, an organism which is difficult to lyse. Protocols developed for *Rhodococcus* sp. strain RHA1, TRIzol, RNA Powersoil Total RNA Isolation Kit (Mo Bio Laboratories, Inc.), and EZNA Bacterial RNA Kit (Omega Bio-Tek, Inc., Norcross, GA, USA) methods did not yield sufficient RNA for sequencing. In the end, sample pellets stored at – 80 °C were thawed on ice and all subsequent steps were performed at 4 °C. Thawed pellets were resuspended in 1.2 mL of RLT buffer (Qiagen); RLT buffer was prepared fresh by addition of 10 µL of β-mercaptoethanol to 1 mL of RLT buffer. To prepare lysis beads, 500 µL of RLT buffer was added to Lysing Matrix B (MP Biomedicals—MP116911050) in 2-mL tubes, followed by vortexing to remove trapped air, then addition of 1.2 mL of bacterial suspension. Bacteria were lysed using a Fastprep-24 classic (MP biomedical), beating at 4 m s^−1^ for 40 s a total of three times with incubation of tubes on ice for 5 min between each bead beating step. The samples were centrifuged for 4 min at 21,130×*g* and supernatant transferred to a fresh 2-mL microcentrifuge tube. It is important to note that the cell debris from lysis of *Gordonia* behaves oddly; cell debris is buoyant and settles between beads and the supernatant. Thus, removal of the supernatant requires very careful pipetting to not disturb the pellet. In the clean tube, an equal amount of pre-chilled 70% ethanol was added to the supernatant and mixed well by pipetting. RNA was isolated and cleaned on an RNeasy Mini (Qiagen) spin column as per manufacturer’s guidelines (RNeasy Mini Kit. ID: 74104); if a sample volume was > 700 µL, the total sample volume was passed serially through a column until all sample was purified. Total RNA was eluted in 30 µL of RNase free water. The concentration of RNA was measured using the Qubit RNA BR kit (Thermo Fisher Scientific).

### DNase I treatment and depletion of ribosomal RNA

RNA samples were treated with Ambion Turbo DNA free kit (Thermo Fisher Scientific) to remove contaminating DNA, following the manufacturer’s instructions. Extracted RNA was stored at − 80 °C. Ribosomal RNA (rRNA) depletion was carried out as per manufacturer’s guidelines using the Gram positive Bacterial Ribo Zero rRNA depletion kit (Illumina MRZGP126). Post depletion the quantity and quality of RNA was checked using Qubit RNA BR kit and the RNA Nano 6000 LabChip kit (Agilent Technologies, USA) following the manufacturer’s instructions.

### Library preparation and sequencing

After rRNA depletion, RNA was converted to DNA for sequencing using the NEBNext Ultra II Directional RNA Library Prep Kit (NEB #E7760). FTAB and OCT samples were sequenced on the Illumina MiSeq platform using the V3 150 cycle paired-end cartridge; 6:2 FTSA and MgSO_4_ samples were sequenced on the Illumina MiniSeq using the 150-cycle paired-end cartridge and MiniSeq 75 cycles protocol, respectively.

### Data analysis and accession numbers

For all libraries, forward read sequence data was processed with Trimmomatic (Bolger et al. 2014) using TruSeq3-SE.fa adapters and the parameters: crop, 75, headcrop 10, leading 3, trailing 3, slidingwindow 4:20, minlen 36. The output was examined in FastQC prior to passing to Salmon (Patro et al. 2017) for indexing (k = 21, p = 8), and quantification (–validateMappings). Bowtie2 (Langmead et al. 2009) was used for alignments (-q -p 8 -X 400 –phred33–very-sensitive) prior to generating counts in HTSeq (Anders et al. 2015) using htseq-count (-m union -s no -i ID -t gene), and passing the SAM file generated by Bowtie2 to SAMtools (Li et al. 2009) to generate a BAM file.

Raw sequencing reads were deposited on NCBI under BioProject PRJNA17734 with the accession numbers: FTAB experiment SRX8382511, run SRR11832043; FTSA experiment SRX8382512, run SRR11832042; OCT experiment SRX8382513, run SRR11832041; MgSO_4_ experiment SRX8382514, run SRR11832040.

Throughout the manuscript, genes are referred to by locus tags from GenBank assembly accession GCA_000347295.2. The corresponding protein identifiers and full annotations for sulfur metabolism, oxygenase, dehydrogenase, oxidoreductase, reductase, CoA transferase and nitrogen metabolism genes can be found in Tables S1 through S4 in the supplemental materials. The corresponding information for genes represented in Fig. 3a and b can be found in Table S5 and Table S6, respectively.

## Results and discussion

### Global gene expression changes for 6:2 FTAB, 6:2 FTSA, OCT and MgSO_4_

**Fig. 1:**
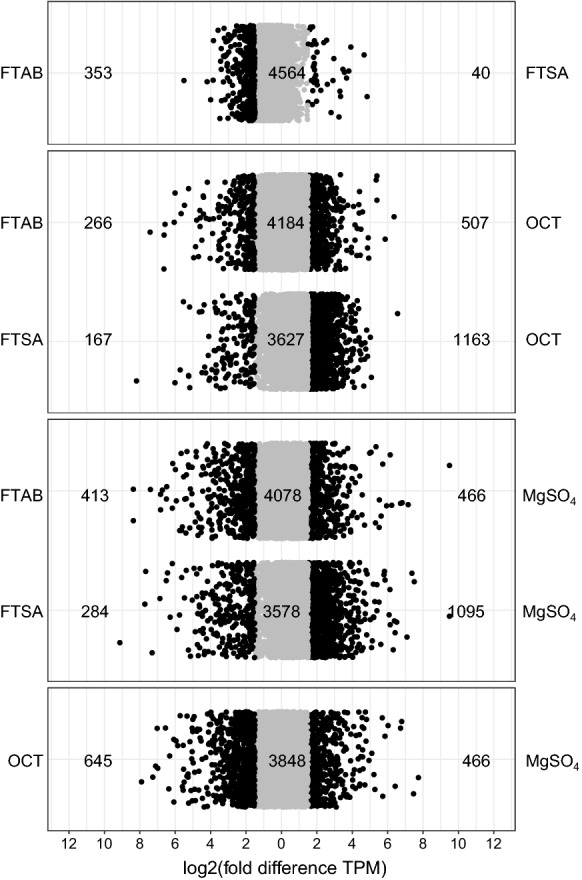
Differences in genome wide gene expression changes when NB4-1Y was provided with 6:2 FTAB, 6:2 FTSA, octanesulfonate (OCT) or MgSO_4_ as sole added sulfur source

To examine how *Gordonia* sp. strain NB4-1Y adapts to using PFAS as a sole source of sulfur for growth, transcriptome sequencing of mid-log phase cultures provided with either 6:2 FTAB, 6:2 FTSA, octanesulfonate (OCT) or MgSO_4_ was carried out. In each case, after NB4-1Y was revived from storage on nutrient agar plates, inoculum cultures were first grown to early stationary phase (approximately six generations; 48 to 72 h depending on the substrate) on 6:2 FTAB, 6:2 FTSA, OCT and MgSO_4_ prior to inoculating experimental cultures and incubating to mid-logarithmic phase (between four and five generations; 40–60 h depending on the substrate). Transcriptional output from each open reading frame was calculated as transcripts per million (TPM) as this is the most robust method for normalizing between culture conditions (Srikumar et al. 2015). Comparing log_2_-fold-differences (log_2_FD) between all combinations of culture conditions show that gene expression patterns were most similar between cells provided with 6:2 FTAB and 6:2 FTSA (Fig. 1), with 393 of 4957 genes (7.9%) expressed ± 1.5 log_2_FD between these two conditions. The greatest difference in transcriptional output was between cultures in OCT compared to MgSO_4_; in this comparison, 2451 (49.4%) genes were expressed ± 1.5 log_2_FD.

**Fig. 2:**
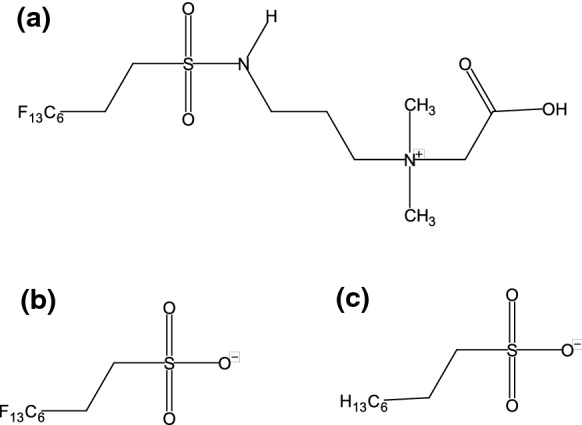
Structures of **a** 6:2 FTAB, **b** 6:2 FTSA, **c** octanesulfonate (OCT)

**Fig. 3:**
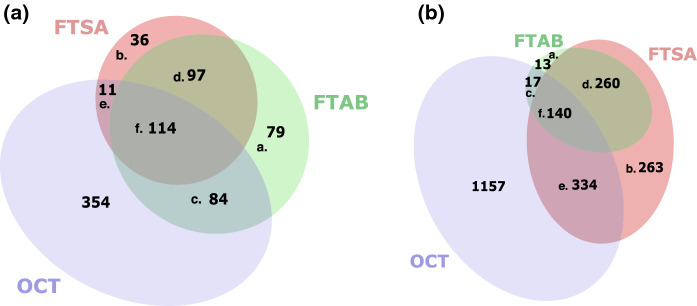
Shared **a** upregulated and **b** downregulated genes for 6:2 FTAB, 6:2 FTSA and octanesulfonate (OCT) as compared to MgSO_4_

Gene expression in both 6:2 FTAB and 6:2 FTSA conditions was more similar to OCT than to MgSO_4_ conditions (Fig. 1), consistent with the structural similarity between OCT, 6:2 FTAB, and 6:2 FTSA. OCT is a non-fluorinated structural analogue of 6:2 FTSA (Fig. 2); 6:2 FTAB and 6:2 FTSA are also structurally very similar, with the former having a sulfonamidoalkyl betaine functional group where the latter has a sulfonate. Owing to the structural similarity of the three metabolites, all subsequent analyses used culture with MgSO_4_ as the reference condition. The consistency between gene expression responses to the two PFAS is illustrated in Venn diagrams examining which genes are more expressed at different levels in 6:2 FTAB and 6:2 FTSA cultures compared to MgSO_4_ cultures. Specifically, 82% of genes showing increased expression in 6:2 FTSA cultures were shared with 6:2 FTAB cultures (Fig. 3a). Similarly, there is significant overlap in genes showing decreased expression in PFAS cultures, with 93% of the genes with reduced expression in 6:2 FTAB cultures also exhibiting reduced expression in 6:2 FTSA cultures (Fig. 3b).

As described below, examination of the putative function of genes with higher levels of expression in PFAS-containing cultures compared to OCT and MgSO_4_ cultures identified genes involved with the transport and metabolism of organosulfur compounds, carbon–nitrogen bond cleavage (in the case of 6:2 FTAB), dehalogenation, formation of acetyl-CoA adducts, and oxidation of the shared C_8_ backbone in 6:2 FTAB and 6:2 FTSA. While changes in gene expression relating to stress in response to PFAS were not detected, putative desulfonation genes that were more highly expressed when NB4-1Y was exploiting 6:2 FTAB and 6:2 FTSA for sulfur were found to code for proteins with reduced cysteine and methionine contents.

Highly expressed genes in the 6:2 FTAB and 6:2 FTSA conditions includes 23 hypothetical proteins and three proteins annotated as ‘domain of unknown function’. Similarly, of the 79 genes more highly expressed upon exposure to 6:2 FTAB, 15 were annotated to code for hypothetical proteins, and one was annotated as ‘domain of unknown function’; for the 36 genes more highly expressed under 6:2 FTSA conditions, there were nine annotated to code for hypothetical proteins and three annotated as ‘domain of unknown function’. These data imply that more detailed analyses in the context of PFAS metabolism may allow us to assign function to genes that are not well annotated in the NB4-1Y genome.

As a control for consistency of core metabolic functions between the four culture conditions, the expression levels of 23 genes associated with a complete citric acid cycle and glyoxylate shunt were examined; these genes would be expected to be similar across treatments given that all cultures had access to the same major carbon sources (acetate and ethanol) under all conditions. There were no striking differences between conditions (Figure S1 in the supplemental materials), with all log_2_FD compared to MgSO_4_ being between − 0.14 and 1.47 (6:2 FTAB 0.97 ± 0.29, 6:2 FTSA 0.78 ± 0.29, OCT 1.08 ± 0.33).

### Sulfur transport and metabolism

**Fig. 4:**
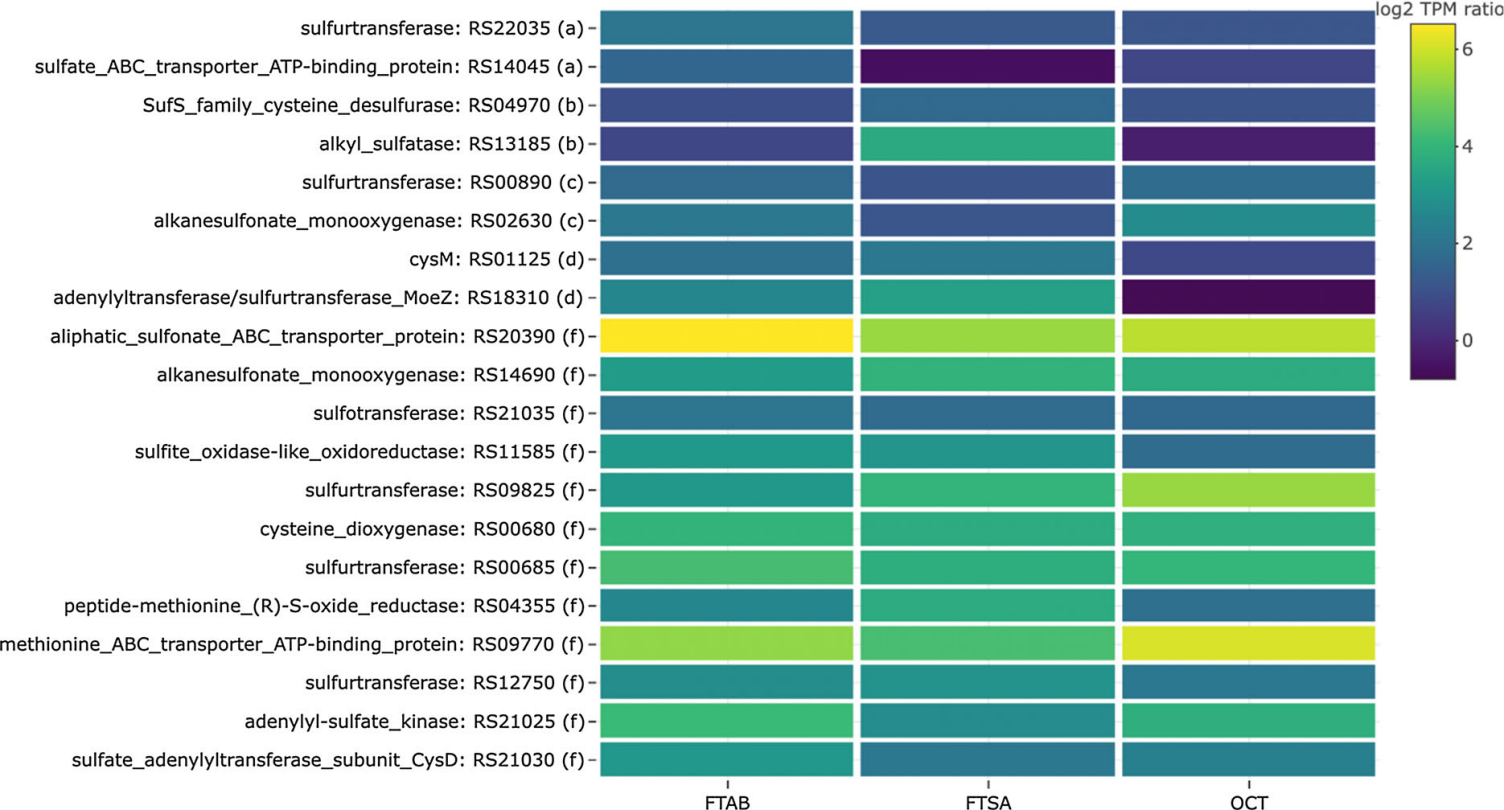
Differential expression levels of sulfur metabolism genes for 6:2 FTAB, 6:2 FTSA and octanesulfonate (OCT) as compared to MgSO_4_. Letters following accession numbers indicate grouping corresponding to those shown in Fig. 3: (a) FTAB; (b) FTSA; (c) FTAB and OCT; (d) FTAB and FTSA; (e) FTSA and OCT; (f) FTAB, FTSA and OCT

NB4-1Y provided with 6:2 FTAB, 6:2 FTSA or OCT are under sulfate limitation, so a global sulfate stress response would be expected (Kertesz 2000; Scott et al. 2007; Zhang et al. 2008). Indeed, 20 genes relating to transport of sulfur compounds, desulfonation and desulfurization, sulfite oxidation, and sulfur transfer were highly expressed across these three conditions as a group when compared to MgSO_4_ (Fig. 4), with some commonalities.

For 6:2 FTAB, a sulfate ABC transporter ATP-binding protein (RS14045) and a sulfurtransferase (RS22035) were more highly expressed when compared to other conditions, while for 6:2 FTSA a cysteine desulfurase (RS04970) and an alkyl sulfatase (RS13185) were more highly expressed. Bacterial sulfate ABC transporters consist of sulfate binding proteins, membrane components, and an ATPase to drive the transport reactions. In *Escherichia coli*, SuBP binds sulfate, CysP binds thiosulfate, CysT and CysW are the membrane components, and CysA is the ATPase (Eitinger et al. 2011). RS14045 in NB4-1Y is adjacent to *cysT*, *cysW*, and a cation transporter gene. Sulfurtransferases and cysteine desulfurases are implicated in the biosynthesis of sulfur-containing biomolecules from cysteine (Kessler 2006), and alkyl sulfatases cleave sulfate ester bonds (Furmanczyk et al. 2018). Given that both 6:2 FTAB and 6:2 FTSA contain a sulfonate sulfur, the higher expression levels of these genes may be related to sulfate starvation rather than specific transport and metabolism of these compounds. Having said that, the alkyl sulfatase may be active against the putatively identified 6:2 FTOH sulfate reported in NB4-1Y cultures provided with 6:2 FTAB or 6:2 FTSA (Shaw et al. 2019).

As noted, in order for NB4-1Y to liberate sulfur from 6:2 FTAB for growth, a number of attack points are available on the sulfonamidoalkyl betaine functional group. The situation is more straightforward with 6:2 FTSA, as the sulfonate is the terminal functional group adjacent to two hydroxylated carbon atoms. Bacterial desulfonation of non-fluorinated sulfonates, such as OCT, has been characterized showing that desulfonation mediated by two-component alkanesulfonate monooxygenase systems (e.g. SsuDE in *E*. *coli*) in the presence of O_2_ and FMNH_2_ generates an aldehyde and sulfite (Zhan et al. 2008). The sulfite would then be chemically or biologically oxidized to sulfate prior to entering reductive sulfate assimilation pathways to generate hydrogen sulfide for incorporation into the amino acid cysteine, from which other sulfur-containing macromolecules such as methionine, iron sulfur cluster containing proteins, thiamin, biotin, and lipoic acid (Kessler 2006) are synthesized. For non-fluorinated substrates, the remaining aldehyde could be oxidized to a carboxylic acid by an aldehyde dehydrogenase prior to the generation, via ß-oxidation, of acetyl-CoA for entry into the citric acid cycle, as is observed in alkane metabolism where an alcohol intermediate precedes the aldehyde (Van Hamme et al. 2003).

We have not observed 6:2 FTAL (aldehyde) in NB4-1Y cultures, either as a product of the 6:2 FTOH that is generated, or as a consequence of an alkanesulfonate monooxygenase-like desulfonation of 6:2 FTSA (Shaw et al. 2019; Van Hamme et al. 2013). However, Martin et al. (2005) noted that 8:2 FTAL was transiently produced as a product of 8:2 FTOH metabolism in rat hepatocytes, being quickly converted to 8:2 FTUAL (unsaturated aldehyde) through non-enzymatic hydrogen fluoride elimination. In order to observe 8:2 FTAL, they had to chemically trap it using 2,4-dinitrophenylhydrazine. 8:2 FTAL was also observed as the first product of 8:2 FTOH metabolism by recombinant human cytochrome P450s, human liver microsomes, and human liver cytosol extracts (Li et al. 2016).

With the exception of a cysteine synthase gene (*cysM*; RS01125) and a sulfurtransferase (RS18310) that were more highly expressed when NB4-1Y was provided either 6:2 FTAB or OCT, the remaining 12 sulfur metabolism genes were more highly expressed with all three substrates when compared to MgSO_4_. These include four sulfurtransferases, a methionine ABC transporter ATP-binding protein, a sulfite oxidase-like oxidoreductase, and a cysteine dioxygenase. An aliphatic sulfonate ABC transport protein (RS20390) was more highly expressed in 6:2 FTAB, 6:2 FTSA and OCT cultures with TPM > 10 (6:2 FTAB, 76.3; 6:2 6:2 FTSA 34.6; OCT, 45.4; MgSO_4_ 0.83). The alkanesulfonate monooxygenase (RS14690) that appears in Fig. 4 passed the log_2_FD threshold, but in all cases the TPM was < 10, suggesting that the gene may not be significantly transcribed and may not be involved in desulfonation of any of the substrates.

**Fig. 5:**
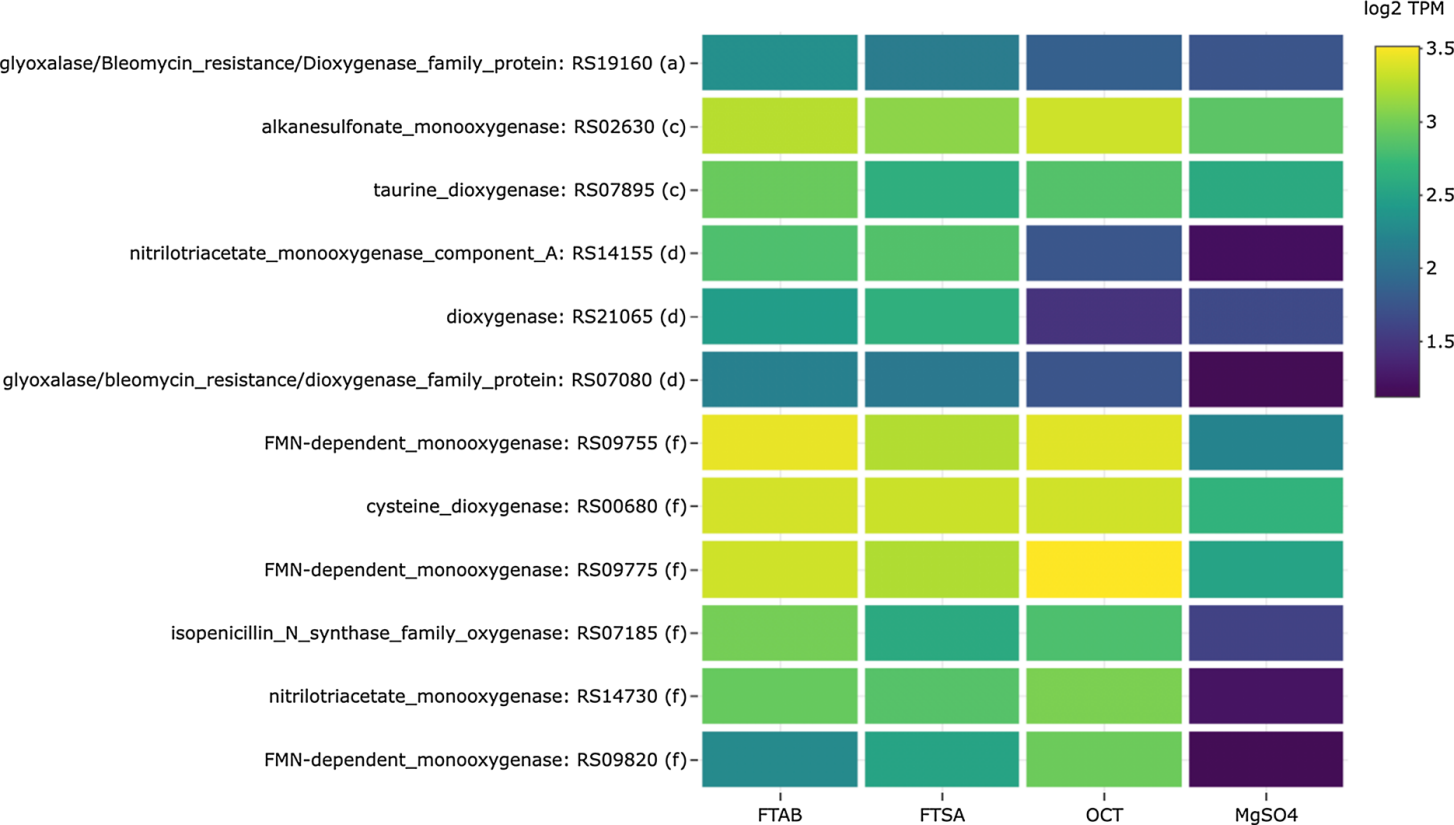
Expression levels of oxygenase genes for 6:2 FTAB, 6:2 FTSA, octanesulfonate (OCT) and MgSO_4_. Letters following accession numbers indicate grouping corresponding to those shown in Fig. 3: (a) FTAB; (b) FTSA; (c) FTAB and OCT; (d) FTAB and FTSA; (e) FTSA and OCT; (f) FTAB, FTSA and OCT

A search for oxygenases with TPM > 10 in the transcriptomic datasets generated a list of 12 genes that, for some conditions, yielded log_2_FD > 1.5 (Fig. 5). Of particular interest is an alkanesulfonate monooxygenase gene (RS02630) that, unlike RS14690 mentioned above, was expressed under all conditions, with the strongest response in 6:2 FTAB and OCT cultures when compared to MgSO_4_ (TPM values: 6:2 FTAB, 756.7; 6:2 FTSA, 369.6; OCT, 1095.8; MgSO_4_, 166.8). Two FMN-dependent monooxygenases, RS09755 and RS09775 (discussed further below), were more highly expressed in 6:2 FTAB, 6:2 FTSA and OCT containing cultures, and the TPM values were higher than for the alkanesulfonate monooxygenase. In addition to these genes, one taurine dioxygenase gene was expressed under all conditions, although the log_2_FD compared to MgSO_4_ was only above 1.5 for 6:2 FTAB, and not for 6:2 FTSA or OCT. Taurine dioxygenases are 2-oxoglutarate-dependent enzymes that cleave 2-aminoethane sulfonate (taurine) to release sulfite, aminoacetaldehyde, succinate and CO_2_, and are active against other organosulfonates including butane-, pentane- and hexanesulfonic acids (Eichhorn et al. 1997).

### Sulfur starvation response

**Fig. 6:**
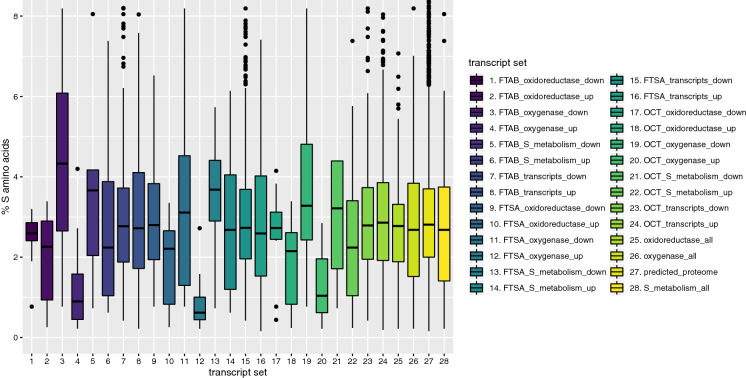
Percent sulfur-containing amino acids in the predicted proteome for up- and down-regulated genes for 6:2 FTAB, 6:2 FTSA and octanesulfonate (OCT) as compared to MgSO_4_

We previously observed in two-dimensional differential in-gel electrophoresis (2D DIGE) experiments that two FMN-dependent monooxygenases (RS09755 and RS09775) that were produced when NB4-1Y was provided with 6:2 FTSA as a sole added source of sulfur, had a lower percentage of sulfur-containing amino acids (0.2 and 0.67%, respectively) than the overall predicted proteome (Van Hamme et al. 2013). It has been broadly observed in bacteria, yeast and algae that proteins involved in sulfur acquisition are lacking in cysteine and methionine to ensure continued growth under sulfur limiting conditions (Mazel and Marliére 1989; Merchant and Helmann 2012). To explore this further in NB4-1Y, the percent sulfur containing amino acids for oxygenase, oxidoreductase and sulfur metabolism proteins associated with genes that were expressed at both higher and lower levels when compared to MgSO_4_, was calculated (Fig. 6). Calculations were also made for all genes that were expressed at both higher and lower levels when compared to MgSO_4_, regardless of function, and for the overall predicted proteome. The most striking trend observed is that the more highly expressed oxygenase genes, when compared to MgSO_4_, for 6:2 FTAB, 6:2 FTSA and OCT, code for proteins with 1.24 (n = 13), 0.88 (n = 10) and 1.33 (n = 11) percent sulfur-containing amino acids, respectively, compared to the overall proteome average of 3.01% (n = 4351). Furthermore, oxygenases that were expressed at lower levels for these three conditions averaged 4.41 (n = 4), 3.48 (n = 8) and 3.82 (n = 7) percent, respectively.

### Carbon–nitrogen bond cleavage

It has been hypothesized that 6:2 FTSA may be a degradation product of 6:2 FTAB (D’Agostino and Mabury 2017), although no direct experimental evidence is available in support of this. Presumably, the sulfonamidoalkyl betaine functional group could be attacked at the terminal carboxyl group, the subterminal methyl groups, by direct attack of the sulfur–nitrogen bond, or cleavage of one of the carbon–nitrogen bonds. Three genes that were expressed only upon NB4-1Y exposure to 6:2 FTAB that may be implicated in carbon–nitrogen bond cleavage are: a carbon–nitrogen hydrolase family protein gene (RS19120: 12.8 TPM compared to between 3.4 and 4.7 for other conditions); an alkaline ceramidase gene (RS02470: 13.7 TPM compared to 0.23–0.36 for other conditions); and an amine oxidase (RS07660: 14.1 TPM compared to between 0.76 and 1.58 for other conditions). Carbon–nitrogen hydrolases are known to liberate ammonia from organic nitrogen compounds (Bork and Koonin 1994; Thuku et al. 2009), and this family of proteins is similar to the characterized R-amidase in *Pseudomonas* sp. MCI3434 which cleaves the carbon–nitrogen bond in (R,S)-piperazine-2-*tert*-butylcarboxamide to form (R)-piperazine-2-carboxylic acid (Komeda et al. 2004). Ceramidases hydrolyze *N*-acyl linkages in ceramides generating sphingosine (2-amino-4-*trans*-octadecene-1,3-diol) and free fatty acids, and have been isolated from Pseudomonads associated with dermatitis in humans (Okino et al. 1998). Bacterial amine oxidases have been described that use molecular oxygen to liberate ammonia, hydrogen peroxide and aldehydes from amines (McGuirl and Dooley 1999; Suzzi and Gardini 2003).

Two nitrilotriacetate monooxygenase genes (RS14730 and RS14155) were more highly expressed in the presence of 6:2 FTAB compared to MgSO_4_, but both of these genes were also expressed in the presence of 6:2 FTSA, and one was expressed in the presence of OCT. Specifically, RS14730 was not expressed when MgSO_4_ was the sole added sulfur source (TPM < 5), and the log_2_FD for 6:2 FTAB, 6:2 FTSA and OCT were 5.37, 4.91 and 5.85, respectively. RS14155 was not expressed with OCT or MgSO_4_ (TPM < 10 for both), and the log_2_FD for 6:2 FTAB and 6:2 FTSA were 4.76 and 4.86, respectively. Nitrilotriacetate monooxygenases convert nitrilotriacetate to iminodiacetate and glyoxylate, and these enzymes are in a subclass of flavoprotein monooxygenases that includes alkanesulfonate monooxygenases (e.g. SsuD) capable of desulfonating a broad range of sulfonated compounds (Eichhorn et al. 1999; Ellis 2010, 2011; Endoh et al. 2003; Kahnert et al. 2000; Koch et al. 2005; Robbins and Ellis 2012; van Berkel et al. 2006; van der Ploeg et al. 1998; Xu et al. 1997). Given this, and our observation that two FMN-dependent monooxygenases first annotated as nitrilotriacetate monooxygenases (RS09755—old locus tag 1222; RS09775—old locus tag 1218) were produced in 6:2 FTSA cultures, and not MgSO_4_ cultures, we hypothesized that this class of enzyme may be able to cleave carbon–sulfur bonds as well as carbon–nitrogen bonds (Van Hamme et al. 2013).

### PFAS backbone metabolism

**Fig. 7:**
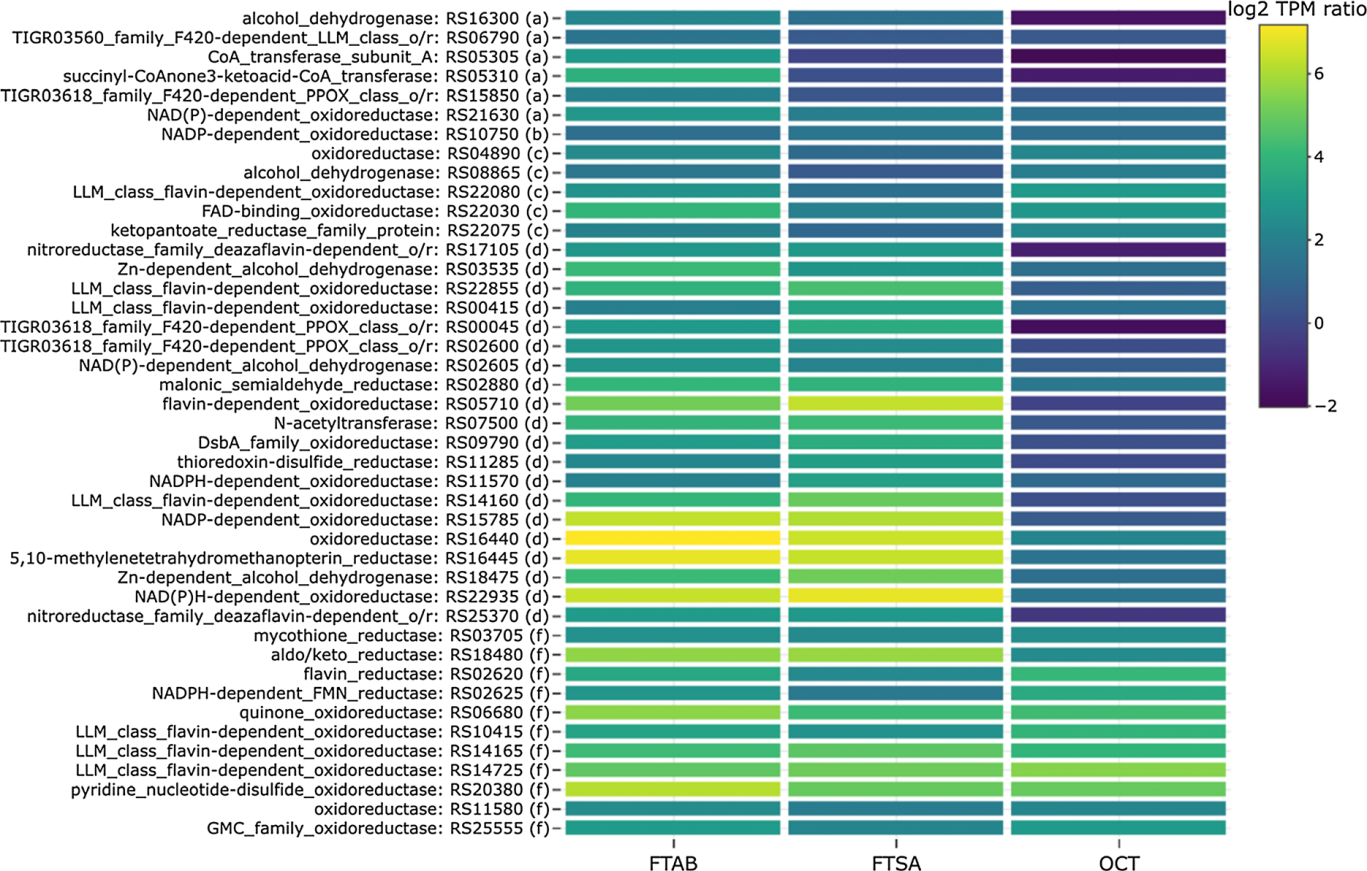
Differential expression levels of dehydrogenase, oxidoreductase, reductase and CoA transferase genes for 6:2 FTAB, 6:2 FTSA and octanesulfonate (OCT) as compared to MgSO_4_. Letters following accession numbers indicate grouping corresponding to those shown in Fig. 3: (a) FTAB; (b) FTSA; (c) FTAB and OCT; (d) FTAB and FTSA; (e) FTSA and OCT; (f) FTAB, FTSA and OCT

Previous analytical studies detected eleven quantifiable breakdown products in NB4-1Y cultures provided with either 6:2 FTAB or 6:2 FTSA (Shaw et al. 2019): perfluorobutanoate (PFBA), perfluoropentanoate (PFPeA), perfluorohexanoate (PFHxA), perfluoroheptanoate (PFHpA), 4:3 fluorotelomer carboxylate (4:3 FTCA), 5:3 FTCA, 6:2 FTCA, 6:2 fluorotelomer unsaturated carboxylate (6:2 FTUA), 5:2 fluorotelomer secondary alcohol (5:2 sFTOH), 5:2 fluorotelomer ketone (5:2 FT ketone), and 6:2 fluorotelomer alcohol (6:2 FTOH). In addition to these, qualitatively identified products included: 4:2 FTCA in cultures containing either 6:2 FTSA and 6:2 FTAB; 4:2 FT ketone in cultures containing 6:2 FTSA; and 4:2 FTUA in cultures containing 6:2 FTAB. Further, 6:2 FTOH sulfate was tentatively identified in both 6:2 FTSA and 6:2 FTAB cultures. In all, for both 6:2 FTAB and 6:2 FTSA, following removal of the functional group, a series of dehalogenation, desaturation, oxidation and decarboxylation reactions are required to shorten the fluorinated backbone. These reactions may be biologically mediated, or may be a combination of biologically mediated and spontaneous chemical reactions. In an effort to identify genes that may be involved in the biological reactions, we searched for dehydrogenase, oxidoreductase, reductase and CoA transferase genes that were highly expressed in 6:2 FTAB, 6:2 FTSA and OCT cultures as compared to MgSO_4_ (Fig. 7).

One alcohol dehydrogenase gene (RS16300) was uniquely expressed in 6:2 FTAB grown cultures, this could be attributed to oxidative attack of the methyl groups in the functional group or to oxidation of the 6:2 FTOH detected as a metabolic intermediate (Shaw et al. 2019). Two alcohol dehydrogenase genes (RS02605 and RS18475) were expressed in 6:2 FTAB and 6:2 FTSA cultures, but not in OCT cultures, and one additional alcohol dehydrogenase (RS08865) was expressed in 6:2 FTAB and OCT cultures.

Following this, three luciferase-like monooxygenase (LLM) class flavin-dependent oxidoreductase genes (RS22855, RS00415, RS14160) were expressed in 6:2 FTAB and 6:2 FTSA cultures. LLMs are flavin-dependent monooxygenases, a group of enzymes that catalyze a diversity of reactions including hydroxylations, epoxidations, and Baeyer–Villiger rearrangements (Maier et al. 2015). The well characterized luciferases that drive bioluminescence reactions in some marine bacteria oxidizes aliphatic aldehydes to carboxylic acids (Ellis 2010). Three additional LLM class flavin-dependent oxidoreductase genes (RS10415, RS14165, RS14725) were expressed in 6:2 FTAB, 6:2 FTSA and OCT cultures, and one more (RS22080) was expressed in 6:2 FTAB and OCT cultures, reflective of the versatility of this enzyme class. In addition to these, two additional oxidoreductases were expressed in 6:2 FTAB cultures, and ten other oxidoreductases and three reductases were uniquely expressed in 6:2 FTAB and 6:2 FTSA cultures, all of which need further examination for potential roles in PFAS metabolism.

During beta oxidation of fatty acids, coenzyme A (CoA) is added to the carboxyl group to yield a fatty acyl-CoA ester from which acetyl-CoA is eventually liberated for entry into the citric acid cycle. While CoA adducts of PFAS have not been reported, other conjugates such as glutathione-8:2 FTUAL have been reported (Martin et al. 2005). In NB4-1Y, one CoA transferase subunit A gene (RS05305) was expressed in 6:2 FTAB cultures, and an *N*-acetyltransferase gene (RS07500) was expressed in 6:2 FTAB and 6:2 FTSA cultures. This may indicate that acetyl-CoA adducts are formed during PFAS metabolism, but are not detected using standard PFAS analytical methods.

### Stress response

With the potential for hydrogen fluoride production in 6:2 FTAB and 6:2 FTSA cultures, NB4-1Y may be exposed to greater than normal oxidative stress during metabolism of these compounds. A collection of 67 stress response genes were examined, including catalase, peroxidase, superoxide dismutase and organic hydroperoxide resistance genes, cold and heat shock genes, universal stress response genes, and DNA repair genes. No major differences in gene expression levels were detected between the four conditions tested (Figure S2), suggesting that NB4-1Y is well suited to thrive in PFAS-contaminated environments. Overall, as for genes associated with the citric acid cycle, no obvious differences between the culture conditions emerged for known stress response genes.

We previously detected the production of an alkyl hydroperoxide reductase (AhpC; now annotated as peroxiredoxin RS04175) when NB4-1Y was exposed to 6:2 FTSA for 2D DIGE experiments, a protein that was not produced when NB4-1Y was grown on MgSO_4_. Alkyl hydroperoxide reductases and peroxiredoxins are part of a robust reactive oxygen species defence system found across Bacteria; the former converts damaging hydroperoxides to alcohols, the latter reduces metabolically generated hydrogen peroxide to water (Johnson and Hug 2019). AhpC has been reported to be a sulfate starvation-induced protein in both *E. coli* and *Pseudomonas* (Kertesz 2000). While, when compared to MgSO_4_, RS04175 showed a higher level of expression under 6:2 FTAB and 6:2 FTSA conditions, it also exhibited a higher expression level with OCT, suggesting that this particular stress response is not linked to fluorinated substrates.

## Conclusion

The data presented here illustrate dynamic and unique gene expression changes in *Gordonia* sp. strain NB4-1Y in response to being provided with 6:2 FTAB or 6:2 FTSA as sole sources of added sulfur for growth. These changes are driven by sulfate starvation conditions that are the norm in natural soil environments (Kertesz 2000), as reflected by the expression of genes associated with organosulfur compound transport, desulfurization of sulfur-containing amino acids, and putative desulfinases with significantly lower percentages of those amino acids. A set of dehydrogenase, oxygenase, oxidoreductase, acetyl-CoA transferase genes, and genes associated with carbon–nitrogen bond cleavage, were also more highly expressed upon exposure of NB4-1Y to 6:2 FTAB and 6:2 FTSA, potentially implicating them in PFAS metabolism. Currently, these genes are being cloned, expressed, and purified, for crystallographic and enzymatic characterization. The end goal is to better understand what specific enzymes are responsible for PFAS breakdown in the environment, and to determine which steps in breakdown pathways are biologically mediated and which are chemically mediated. These data may be useful for developing quantitative polymerase chain reaction screening panels to monitor passive and active in situ bioremediation efforts for water, soil and groundwater. Additionally, as gene and protein functions are elucidated, the data will inform multi-omic studies of PFAS-impacted environments, and through the improved RNA extraction methods described here, potentially reveal that *Gordonia* are underrepresented in environmental metatranscriptomes.

## Electronic supplementary material

Below is the link to the electronic supplementary material.

Supplementary file1 (DOCX 656 KB)

Supplementary file2 (XLSX 80 KB)

Supplementary file3 (XLSX 210 KB)

Supplementary file4 (PDF 576 KB)
