# Supplementary material for: Transcriptomic response of Gordonia sp. strain NB4-1Y when provided with 6:2 fluorotelomer sulfonamidoalkyl betaine or 6:2 fluorotelomer sulfonate as sole sulfur source
Source: Biodegradation. 2020 Nov 5;31(4):407–22. doi: 10.1007/s10532-020-09917-8 (PMC7661421; doi:10.1007/s10532-020-09917-8)
Supplement: Supplementary file 1 — Supplementary file1 (DOCX 656 KB) [file 10532_2020_9917_MOESM1_ESM.docx]

**Supplementary Material**

**Transcriptomic response of *Gordonia* sp. strain NB4-1Y when provided with 6:2 fluorotelomer sulfonamidoalkyl betaine or 6:2 fluorotelomer sulfonate as sole sulfur source**

Eric M. Bottos^a^, E.Y. AL-shabib^b,c^, Dayton M.J. Shaw^a^, Breanne M. McAmmond^a^, A. Sharma^b,c^, D.M. Suchan^b,c^, Andrew D.S. Cameron^b,c^, Jonathan D. Van Hamme^a,^*

^a^ Department of Biological Sciences, Thompson Rivers University, Kamloops, BC V2C 0C8, Canada

^b^ Department of Biology, University of Regina, Regina, SK S4S 0A2

^c^ Institute for Microbial Systems and Society, Faculty of Science, University of Regina, Regina, SK S4S 0A2

*Corresponding author: [jvanhamme@tru.ca](mailto:jvanhamme@tru.ca) (J.D. Van Hamme)

ORCID IDs:

E.M. Bottos 0000-0001-9671-5657

E.Y. AL-shabib 0000-0001-6860-7931

B.M. McAmmond 0000-0001-5370-6152

A. Sharma 0000-0001-9982-5122

D.M. Suchan 0000-0001-8961-652X

A.D.S Cameron 0000-0003-1560-8572

J.D. Van Hamme 0000-0001-9471-7616

**Contents**

10 Pages

02 Figures

04 Tables

**Table of Contents**

[FIGURES 3](#_Toc41045604)

[TABLES 6](#_Toc41045605)

# FIGURES

**Figure S1.** Expression levels of genes associated with the tricarboxylic acid cycle for 6:2 FTAB, 6:2 FTSA, octanesulfonate (OCT) and MgSO_4_.

**
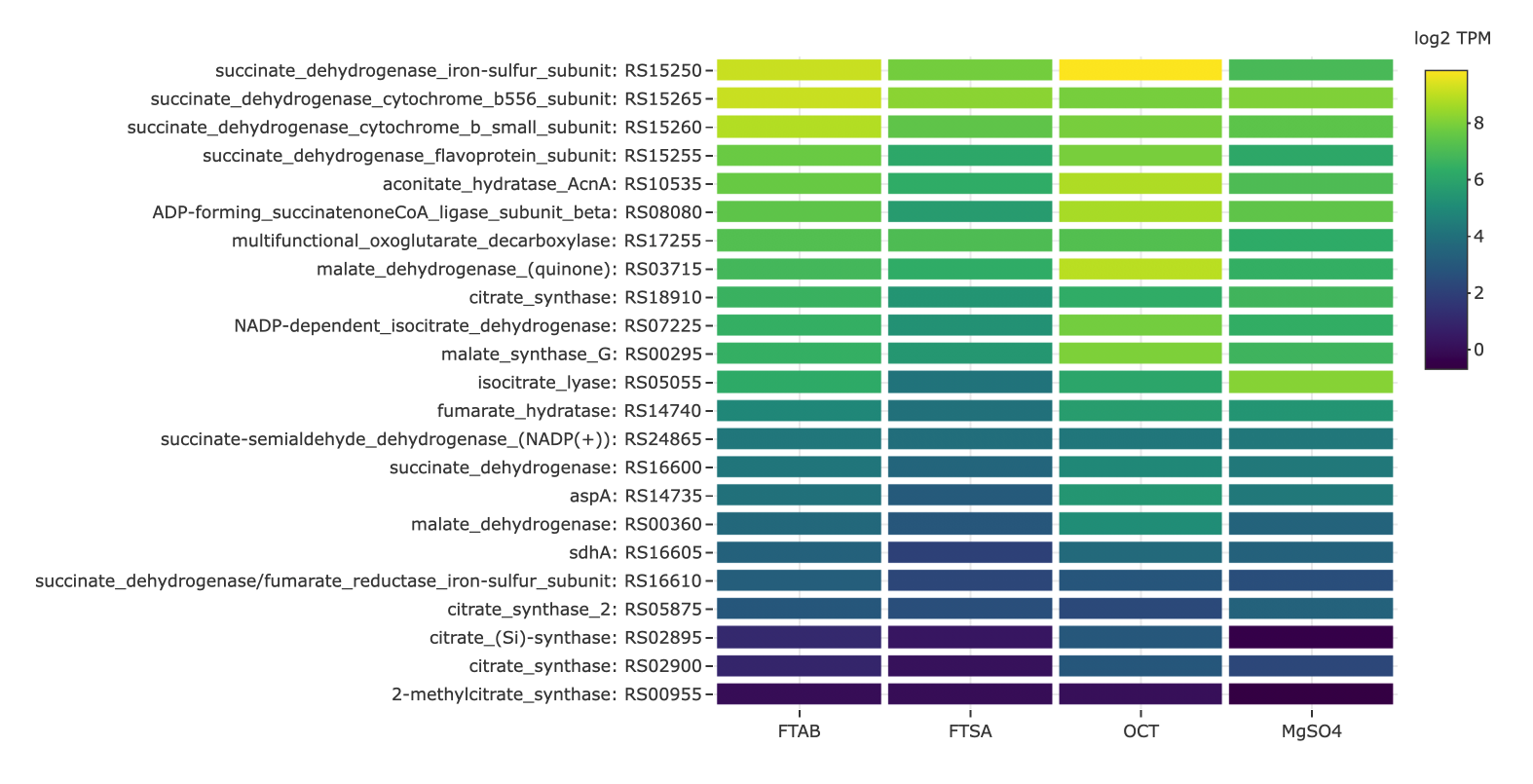
**

**Figure S2.** Expression levels of genes associated with stress response for 6:2 FTAB, 6:2 FTSA, octanesulfonate (OCT) and MgSO_4_.

**
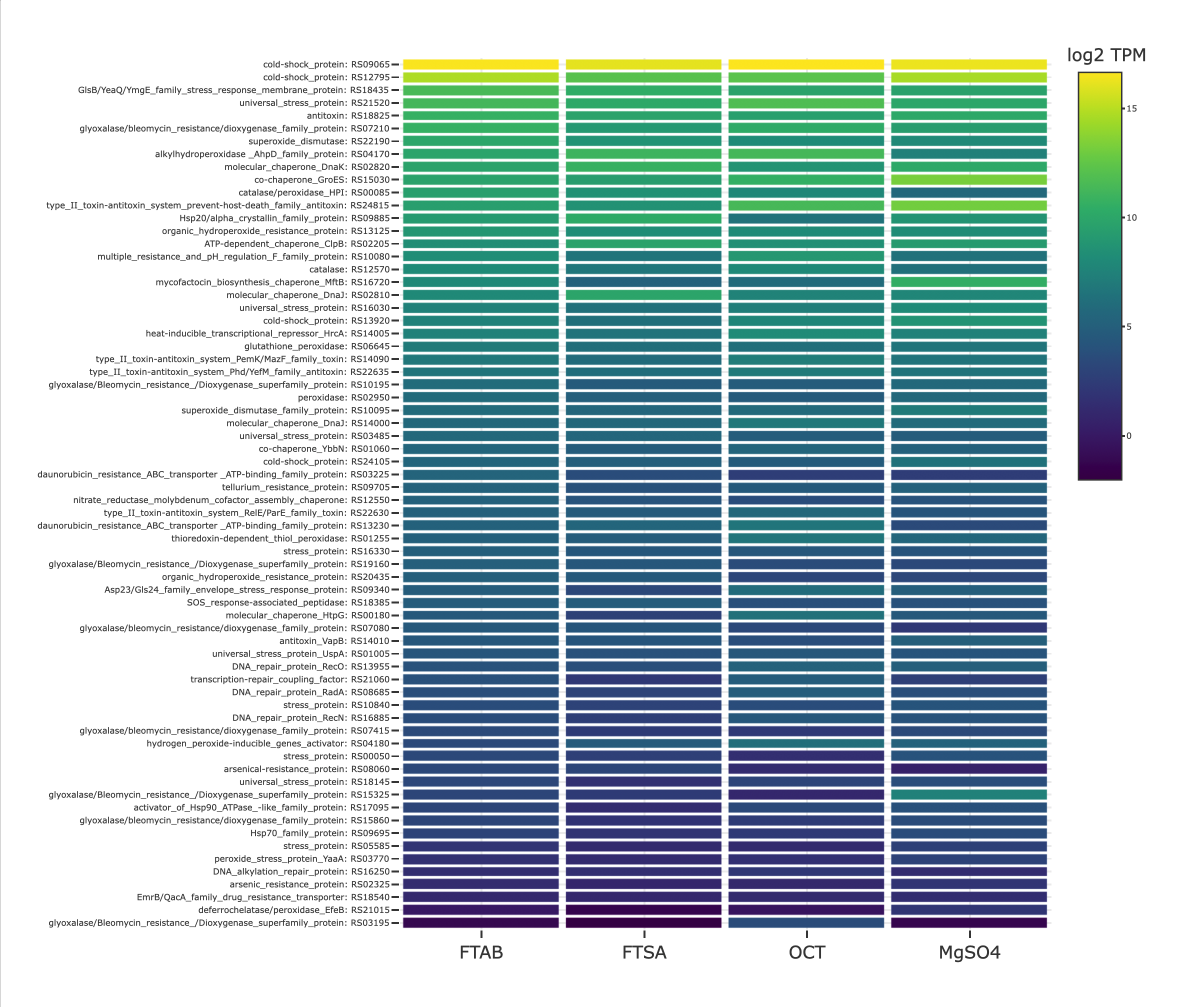
**

# TABLES

**Table S1.** Locus tags, protein identifier numbers and annotations for sulfur metabolism genes presented in Figure 3.

| locus_tag | protein_id | annotation |  |
| --- | --- | --- | --- |
| ISGA_RS00680 | WP_053776057.1 | cysteine_dioxygenase_CDS |  |
| ISGA_RS00685 | WP_053776062.1 | sulfurtransferase_CDS |  |
| ISGA_RS00890 | WP_053776084.1 | sulfurtransferase_CDS |  |
| ISGA_RS01125 | WP_020792888.1 | cysM_CDS |  |
| ISGA_RS02630 | WP_053776271.1 | alkanesulfonate_monooxygenase_CDS |  |
| ISGA_RS04355 | WP_053776448.1 | peptide-methionine_(R)-S-oxide_reductase_CDS |  |
| ISGA_RS04970 | WP_020790814.1 | SufS_family_cysteine_desulfurase_CDS |  |
| ISGA_RS09770 | WP_053777104.1 | methionine_ABC_transporter_ATP-binding_protein_CDS |  |
| ISGA_RS09825 | WP_020793849.1 | sulfurtransferase_CDS |  |
| ISGA_RS11585 | WP_020793028.1 | sulfite_oxidase-like_oxidoreductase_CDS |  |
| ISGA_RS12750 | WP_053777439.1 | sulfurtransferase_CDS |  |
| ISGA_RS13185 | WP_053777484.1 | alkyl_sulfatase_CDS |  |
| ISGA_RS14045 | WP_020792788.1 | sulfate_ABC_transporter_ATP-binding_protein_CDS |  |
| ISGA_RS14690 | WP_020790493.1 | alkanesulfonate_monooxygenase_CDS |  |
| ISGA_RS18310 | WP_053778120.1 | adenylyltransferase/sulfurtransferase_MoeZ_CDS |  |
| ISGA_RS20390 | WP_020790120.1 | aliphatic_sulfonate_ABC_transporter_periplasmic_ligand-binding_protein_CDS | |
| ISGA_RS21025 | WP_053778428.1 | adenylyl-sulfate_kinase_CDS |  |
| ISGA_RS21030 | WP_053778449.1 | sulfate_adenylyltransferase_subunit_CysD_CDS |  |
| ISGA_RS21035 | WP_020792363.1 | sulfotransferase_CDS |  |
| ISGA_RS22035 | WP_020792334.1 | sulfurtransferase_CDS |  |

**Table S2.** Locus tags, protein identifier numbers and annotations for oxygenase genes presented in Figure 4.

| locus_tag | protein_id | annotation |
| --- | --- | --- |
| ISGA_RS00680 | WP_053776057.1 | cysteine_dioxygenase |
| ISGA_RS02630 | WP_053776271.1 | alkanesulfonate_monooxygenase |
| ISGA_RS07080 | WP_053776782.1 | glyoxalase/bleomycin_resistance/dioxygenase_family_protein |
| ISGA_RS07185 | WP_053776834.1 | isopenicillin_N_synthase_family_oxygenase |
| ISGA_RS07895 | WP_053776880.1 | taurine_dioxygenase |
| ISGA_RS09755 | WP_020794796.1 | FMN-dependent_monooxygenase |
| ISGA_RS09775 | WP_053777105.1 | FMN-dependent_monooxygenase |
| ISGA_RS09820 | WP_020793848.1 | FMN-dependent_monooxygenase |
| ISGA_RS14155 | WP_020789707.1 | nitrilotriacetate_monooxygenase_component_A |
| ISGA_RS14730 | WP_053777671.1 | nitrilotriacetate_monooxygenase |
| ISGA_RS19160 | WP_020793361.1 | glyoxalase/Bleomycin_resistance/Dioxygenase_superfamily_protein |
| ISGA_RS21065 | WP_053778432.1 | dioxygenase |

**Table S3.** Locus tags, protein identifier numbers and annotations for dehydrogenase, oxidoreductase, reductase and CoA transferase genes presented in Figure 5.

| locus_tag | protein_id | annotation |
| --- | --- | --- |
| ISGA_RS00045 | WP_053776028.1 | TIGR03618_family_F420-dependent_PPOX_class_oxidoreductase_CDS |
| ISGA_RS00415 | WP_053776025.1 | LLM_class_flavin-dependent_oxidoreductase_CDS |
| ISGA_RS02600 | WP_053776267.1 | TIGR03618_family_F420-dependent_PPOX_class_oxidoreductase_CDS |
| ISGA_RS02605 | WP_053776268.1 | NAD(P)-dependent_alcohol_dehydrogenase_CDS |
| ISGA_RS02620 | WP_020794112.1 | flavin_reductase_CDS |
| ISGA_RS02625 | WP_053776270.1 | NADPH-dependent_FMN_reductase_CDS |
| ISGA_RS02880 | WP_053776360.1 | malonic_semialdehyde_reductase_CDS |
| ISGA_RS03535 | WP_020791709.1 | Zn-dependent_alcohol_dehydrogenase_CDS |
| ISGA_RS03705 | WP_020791537.1 | mycothione_reductase_CDS |
| ISGA_RS04890 | WP_053776497.1 | oxidoreductase_CDS |
| ISGA_RS05305 | WP_053776577.1 | CoA_transferase_subunit_A_CDS |
| ISGA_RS05310 | WP_053776608.1 | succinyl-CoAnone3-ketoacid-CoA_transferase_CDS |
| ISGA_RS05710 | WP_053776627.1 | flavin-dependent_oxidoreductase_CDS |
| ISGA_RS06680 | WP_053776738.1 | quinone_oxidoreductase_CDS |
| ISGA_RS06790 | WP_020791645.1 | TIGR03560_family_F420-dependent_LLM_class_oxidoreductase_CDS |
| ISGA_RS07500 | WP_053776818.1 | N-acetyltransferase_CDS |
| ISGA_RS08865 | WP_053777000.1 | alcohol_dehydrogenase_CDS |
| ISGA_RS09790 | WP_053777108.1 | DsbA_family_oxidoreductase_CDS |
| ISGA_RS10415 | WP_053777181.1 | LLM_class_flavin-dependent_oxidoreductase_CDS |
| ISGA_RS10750 | WP_053777223.1 | NADP-dependent_oxidoreductase_CDS |
| ISGA_RS11285 | WP_053777296.1 | thioredoxin-disulfide_reductase_CDS |
| ISGA_RS11570 | WP_053777311.1 | NADPH-dependent_oxidoreductase_CDS |
| ISGA_RS11580 | WP_082365884.1 | oxidoreductase_CDS |
| ISGA_RS14160 | WP_053777639.1 | LLM_class_flavin-dependent_oxidoreductase_CDS |
| ISGA_RS14165 | WP_053777622.1 | LLM_class_flavin-dependent_oxidoreductase_CDS |
| ISGA_RS14725 | WP_053777670.1 | LLM_class_flavin-dependent_oxidoreductase_CDS |
| ISGA_RS15785 | WP_053777820.1 | NADP-dependent_oxidoreductase_CDS |
| ISGA_RS15850 | WP_053777873.1 | TIGR03618_family_F420-dependent_PPOX_class_oxidoreductase_CDS |
| ISGA_RS16300 | WP_020789492.1 | alcohol_dehydrogenase_CDS |
| ISGA_RS16440 | WP_053777909.1 | oxidoreductase_CDS |
| ISGA_RS16445 | WP_053777910.1 | 5,10-methylenetetrahydromethanopterin_reductase_CDS |
| ISGA_RS17105 | WP_020789556.1 | nitroreductase_family_deazaflavin-dependent_oxidoreductase_CDS |
| ISGA_RS18475 | WP_053778136.1 | Zn-dependent_alcohol_dehydrogenase_CDS |
| ISGA_RS18480 | WP_020791569.1 | aldo/keto_reductase_CDS |
| ISGA_RS20380 | WP_053778352.1 | pyridine_nucleotide-disulfide_oxidoreductase_CDS |
| ISGA_RS21630 | WP_053778502.1 | NAD(P)-dependent_oxidoreductase_CDS |
| ISGA_RS22030 | WP_053778555.1 | FAD-binding_oxidoreductase_CDS |
| ISGA_RS22075 | WP_082366096.1 | ketopantoate_reductase_family_protein_CDS |
| ISGA_RS22080 | WP_053778553.1 | LLM_class_flavin-dependent_oxidoreductase_CDS |
| ISGA_RS22855 | WP_020793433.1 | LLM_class_flavin-dependent_oxidoreductase_CDS |
| ISGA_RS22935 | WP_053778656.1 | NAD(P)H-dependent_oxidoreductase_CDS |
| ISGA_RS25370 | WP_082365981.1 | nitroreductase_family_deazaflavin-dependent_oxidoreductase_CDS |
| ISGA_RS25555 | WP_082366093.1 | GMC_family_oxidoreductase_CDS |

**Table S4.** Locus tags, protein identifier numbers and annotations for nitrogen metabolism genes.

| locus_tag | protein_id | annotation |
| --- | --- | --- |
| ISGA_RS02470 | WP_053776248.1 | alkaline_ceramidase_CDS |
| ISGA_RS19120 | WP_053778208.1 | carbon-nitrogen_hydrolase_CDS |
